# Editorial: The complexity of urticaria

**DOI:** 10.3389/falgy.2023.1149540

**Published:** 2023-02-17

**Authors:** Michael Makris, Sabine Altrichter, Luis Felipe Ensina, Emek Kocatürk, Michael Rudenko

**Affiliations:** ^1^ Allergy Unit, 2nd Department of Dermatology and Venereology, Medical School, National and Kapodistrian University of Athens, Attikon University Hospital, Athens, Greece; ^2^ Institute for Allergology, Charité – Universitätsmedizin Berlin, Corporate Member of Freie Universität Berlin and Humboldt-Universitätzu Berlin, Berlin, Germany; ^3^ Department of Dermatology and Venerology, Kepler University Hospital, Linz, Austria; ^4^ Division of Allergy, Clinical Immunology and Rheumatology, Department of Pediatrics, Federal University of São Paulo, São Paulo, Brazil; ^5^ Department of Dermatology, Koç University School of Medicine, Istanbul, Turkey; ^6^ London Allergy and Immunology Centre, London, United Kingdom

**Keywords:** urticaria, comorbidity, pregnancy, anaphylaxis, urticarial vasculitis

**Editorial on the Research Topic** The complexity of urticaria

Urticaria is a common condition that presents with transient, pruritic wheals, angioedema or both. It often leads to a reduced quality of life and significant socioeconomic burden. Although the definition is clear, characterizing the lesions may be challenging for the health-care provider and an incorrect diagnosis may directly impact in treatment outcomes. Goméz et al. discuss the urgency in improving the diagnostic criteria, and the importance of identifying and managing properly the disease to reduce its burden.

Distinguishing between wheals and urticarial lesions is the first step to provide a correct diagnosis. As important as the characterization of the lesions is the presence of systemic symptoms such as fever, malaise, and arthralgia. Matos et al. propose two interesting diagnostic algorithms and a practical review of acute and chronic urticaria differential diagnosis. Not limited to skin lesions, the authors also review the main features and differences of histaminergic and bradykinin-mediated angioedema, highlighting the importance of an early diagnosis of hereditary angioedema.

Furthermore, according to the international EAACI/GA²LEN/EuroGuiDerm/APAAACI guideline the prevalence of acute urticaria in a lifetime is around 20% (1). The underlying mechanism is associated with degranulation of skin mast cells while the generalized mast cell and basophil degranulation causes anaphylaxis. Besides, acute urticaria and angioedema present as the most prevalent symptoms from the skin in anaphylaxis; thus, in Emergencies and in primary health care setting the differential diagnosis among anaphylaxis and acute urticaria/angioedema is confusing and puzzling in many cases, leading to errors and delayed treatment as adrenaline is the first-line treatment for anaphylaxis, but not for acute urticaria, where H1-antihistamines are the first choice. Ensina et al. provide a comprehensive review on main aspects, similarities and differences regarding definitions, mechanisms, causes, diagnosis and treatment of acute urticaria and anaphylaxis.

Chronic urticaria is not only more common but is also more severe in females, and although it is a relatively common disease, there is not enough information about the effects of hormonal conditions on the disease. Publications from the PREG-CU project showed that urticaria gets better during pregnancy in half of women while one third of the patients experience symptoms' worsening and 10% reported visits to emergency departments due to urticaria exacerbations (2). Almost 60% of pregnant women used medication for urticaria regardless of the trimester they were. Globally, patients with chronic urticaria did not have an increased risk for preterm birth or neonatal problems, except for increased cesarean section frequency, which is most probably associated with the comorbidities of the patients. However, emergency referrals for urticaria exacerbations increased preterm birth risk which emphasizes the importance of keeping urticaria under control during pregnancy. Kocatürk et al. review the reported effects of sex hormones and pregnancy-specific immunological changes on urticaria, the impact of pregnancy on urticaria, and current information and guidance on the management of urticaria during pregnancy and lactation.

The prevalent fear of patients suffering from chronic urticaria and a major concern of treating physicians is the possible co-existence of other severe diseases, especially malignancies and systemic autoimmune disorders. Accordingly, extensive laboratory testing is being performed in many cases in contrary to current evidence and international guidelines (1). Autoimmune, psychiatric, and atopic diseases are the most frequently reported comorbidities among CU patients, while malignancies, cardiovascular and other diseases have also been reported as associated diseases in patients with chronic urticaria although existing data refers to specific populations (3). Papapostolou et al. overview current data on comorbidities of CU, and furthermore comment on the potential linked pathways underlying these diseases. In the era of tailored made intervention, CU patients should be recognized and treated as a multimorbid group with treatment interventions targeting the comorbidities and the urticaria management *per se* until the complete unravelling of the underlying pathophysiology of chronic urticaria.

Despite the fact, that chronic urticaria is a common disease, some inducible forms of chronic urticaria are rare and their diagnosis is often delayed. In a survey conducted in german speaking countries Altrichter et al. reported a marked average diagnostic delay of almost 3 years. Diagnostic provocations and/or laboratory tests were performed in a small minority of patients. Despite several physician contacts 90% of the patients stated to have an uncontrolled disease, resulting in a strong impact on their everyday activities, sleep, and QoL.

Omalizumab is recommended as second-line therapy in chronic spontaneous urticaria. In a Colombian study conducted by Garcia-Gomez et al. 123 patients were followed upon their treatment response. The percentage of patients with controlled CSU at 3 months on omalizumab treatment was 80% and at 6 months 87% respectively, while the safety profile was almost excellent. On the other hand, omalizumab is a comparatively high-cost medicine and access to this treatment can be challenging. Ridge et al. report a dramatic reduction in unplanned healthcare interactions at primary care and emergency departments in Ireland when patients are treated with omalizumab; thus, the increased cost of omalizumab may at least partly counter-balanced by the reduced use of health system resources.

Urticarial vasculitis is a small-vessel leukocytoclastic vasculitis characterized by different clinical manifestations ranging from long-lasting urticarial lesions to severe and potentially life-threatening multi-organ involvement. Petrelli et al. report their experience on 6 patients with refractory normocomplementemic urticarial vasculitis successfully treated with omalizumab suggesting that this biological therapy may be a safe and effective therapeutic option in urticarial vasculitis.

In conclusion, the scientific works of this Research Topic cover different aspects of urticaria and provides state-of-the-art knowledge in the field of urticaria that could be implemented in both recearch projects and clinical practice.
